# Biomarkers Utility: At the Borderline between Cardiology and Neurology

**DOI:** 10.3390/jcdd8110139

**Published:** 2021-10-25

**Authors:** Adriana Ion, Celina Stafie, Ovidiu Mitu, Cosmina Elena Ciobanu, Dan Iliescu Halitchi, Alexandru Dan Costache, Cezara Bobric, Roxana Troase, Ivona Mitu, Bogdan Huzum, Stefania Teodora Duca, Irina Iuliana Costache

**Affiliations:** 1 1st Medical Department, University of Medicine and Pharmacy “Grigore T. Popa”, 700115 Iasi, Romania; adriana.ion@hotmail.com (A.I.); mituovidiu@yahoo.co.uk (O.M.); iliescud@gmail.com (D.I.H.); adcostache@yahoo.com (A.D.C.); stefaniateodoraduca@gmail.com (S.T.D.); ii.costache@yahoo.com (I.I.C.); 2 Cardiology Clinic, Clinical Emergency Hospital “Sf. Spiridon”, 700111 Iasi, Romania; c.elenacosmina@yahoo.com; 3 Department of Preventive Medicine and Interdisciplinarity, University of Medicine and Pharmacy “Grigore T. Popa”, 700115 Iasi, Romania; 4 Neurology Clinic, Clinical Rehabilitation Hospital, 700661 Iasi, Romania; cezara.corbu@yahoo.com (C.B.); roxana.litu@gmail.com (R.T.); 5 Department of Morpho-Functional Sciences II, University of Medicine and Pharmacy “Grigore T. Popa”, 700115 Iasi, Romania; ivonamitu@gmail.com; 6 Department of Physiology, University of Medicine and Pharmacy “Grigore T. Popa”, 700115 Iasi, Romania; bogdan.huzum93@gmail.com

**Keywords:** biomarkers, cardiology, neurology, troponin, cardiovascular diseases, BNP, stroke

## Abstract

Biomarkers are important diagnostic and prognostic tools as they provide results in a short time while still being an inexpensive, reproducible and accessible method. Their well-known benefits have placed them at the forefront of research in recent years, with new and innovative discoveries being implemented. Cardiovascular and neurological diseases often share common risk factors and pathological pathways which may play an important role in the use and interpretation of biomarkers’ values. Among the biomarkers used extensively in clinical practice in cardiology, hs-TroponinT, CK-MB and NTproBNP have been shown to be strongly influenced by multiple neurological conditions. Newer ones such as galectin-3, lysophosphatidylcholine, copeptin, sST2, S100B, myeloperoxidase and GDF-15 have been extensively studied in recent years as alternatives with an increased sensitivity for cardiovascular diseases, but also with significant results in the field of neurology. Thus, given their low specificity, the values interpretation must be correlated with the clinical judgment and other available investigations.

## 1. Introduction

Biomarkers are indicators of functionality for different organs and systems. They exist in the forms of proteins, enzymes or hormones with a changing concentration value depending on certain physiological or pathological conditions. In cardiac diseases, they have proved their usefulness in many situations, and continuous research is still being conducted. The study of biomarkers began in 1954, when enzymes were used for an early diagnosis of myocardial infarction [1]. Subsequent discoveries and technological development have led to their use in many areas of cardiovascular medicine, in particular in heart failure (HF) and ischemia. Neurological pathology often coexists with cardiovascular diseases due to the common risk factors, which may interfere with the values and interpretation of biomarkers, therefore, if one is not aware of the multiple and intricate interpretations, medical judgement errors could result. In this context, we considered it useful to compare and analyze most of the biomarkers studied and used in recent years in order to increase awareness and understanding.

We started by exploring the role of classical biomarker, that are frequently used in clinical practice in cardiology and neurology. Subsequently, we analyzed some new biomakers with great potential for clinical use, but that have not yet been introduced in guidelines and daily practice, as they require more studies (Table 1).

## 2. Biomarkers

### 2.1. Natriuretic Peptides

The heart normally secretes hormones from the atrial tissue for the regulation of homeostasis and blood pressure. The atrial natriuretic peptide (ANP) and the natriuretic peptide type B (BNP) are secreted in response to atrial distension. The integration of biomarkers in heart failure evaluation, including BNP and NT-proBNP (N-terminal pro-hormone BNP), is part of numerous studies on diagnosis and prognostic assessment. The development of biomarkers in the last decade promoted BNP usage in a variety of studies [2].

BNP and NT-proBNP are released from the atrial and ventricular myocardium after the proBNP precursor is cleaved in these two forms in response to the cardiomyocyte stimulation of volume or pressure overload and myocardial ischemia. BNP is an active hormone with vasodilating and diuretic effects that reduces the left ventricular load. NT-proBNP is an inactive fragment, with a half-life greater than that of BNP. The ANP is initially synthesized at the level of the atrial myocardium and its plasma concentration is approximately 5000 times higher than that of BNP [3]. As they reflect volume overload, natriuretic peptides may have elevated concentration due to several cardiac or non-cardiac causes: heart failure (HF), acute coronary syndrome (ACS), pulmonary embolism, valvular heart diseases, pulmonary hypertension, tachyarrhythmias, ischaemic stroke, advanced age, renal dysfunction, liver dysfunction, anaemia, etc [4,5].

High levels of natriuretic peptides correlate with severe symptoms and have a predictive role for morbidity and mortality in heart failure. In cardiac amyloidosis, the plasma level of BNP is three to five times higher than in any other etiology of hypertrophic cardiomyopathy, although the values of the ventricular wall thickness are comparable. In primary amyloidosis, many patients have renal impairment, keeping in mind that the cut-off values need to be adjusted. Both BNP and NT-proBNP are independent prognostic factors in cardiac amyloidosis patients, but BNP showed superior prognostic value in patients that were also associated with end stage kidney disease, as the impact of reduced eGFR was greater on NT-proBNP than on BNP [6]. In patients with Anderson-Fabry heart disease, NT-proBNP correlates with the echocardiographic abnormalities of left ventricular filling, but abnormal peptide levels are also observed in patients without left ventricular hypertrophy, suggesting that natriuretic peptides may be useful in identifying patients with early subclinical disease [7].

The proBNP/BNP ratio may be useful in distinguishing between constrictive pericarditis and restrictive cardiomyopathy. Relatively normal BNP values in patients with right heart failure should raise the suspicion of constrictive pericarditis [8].

BNP and NT-proBNP are among the most intensively studied biomarkers, being recently introduced as part of the stroke risk and hemorrhage assessment in patients with atrial fibrillation. Various data have shown superior results of the troponin and brain natriuretic peptide levels compared to the risk scores recommended in the current guidelines. However, more studies are needed to confirm the position of biomarkers in determining the risk of stroke and bleeding in patients diagnosed with atrial fibrillation [9].

Despite the advances in medical research, cardiogenic stroke remains a health problem with a high mortality rate. Cardiac biomarkers have been reported to be useful indicators for cardiogenic stroke and secondary cardiovascular events. BNP and NT-proBNP levels are strongly correlated with the transthoracic or transesophageal echocardiography parameters of left atrial and left appendage, indicating that they may reflect their dysfunction in stroke patients [3].

Both BNP and NT-proBNP levels are more useful predictors of cardiogenic stroke and major adverse cardiovascular and cerebrovascular events compared to ANP and hsTnT in patients diagnosed with stroke [10]. Although the natriuretic peptide should be carefully used as a prognostic marker for stroke because it lacks optimal baseline values, both BNP and NT-proBNP were very reliable and clinically useful, with a high predictive value regarding the risk for cardiogenic stroke and future cerebrovascular events [3].

Transient ischemic attacks (TIAs) represent 11% of all forms of ischemic stroke. They are defined by the presence of focal arterial ischemia with transient symptoms, with no signs of clinical or imagistical infarction. The importance of TIA is that of predicting cerebral and myocardial infarction, which can be prevented if appropriate therapeutic measures are taken [11].

Serum levels of NT-proBNP greater than 800pg/mL measured in the first hours after TIA are associated with an increased risk of ischemic or hemorrhagic stroke. This biomarker can be a simple and extremely useful alternative to predicting the prognosis after TIA, regardless of the etiopathogenesis of TIA [9].

Highly sensitive NT-proBNP and troponin I are independently associated with dementia and NT-proBNP with Alzheimer’s disease. Furthermore, NT-proBNP is associated with dementia independent of the highly sensitive troponin I value, but in association with the highly sensitive troponin I no significant result was obtained. No differences were observed between these biomarkers when reclassifying the risk of dementia. NT-proBNP may improve risk discrimination in Alzheimer’s disease [12].

### 2.2. Troponins

Troponins (Tn) are cardiac regulatory proteins that intermediate the interaction between thick-filaments (myosin) and thin-filaments (actine and tropomyozine) from the sarcomere through calcium binding, leading to muscle contraction. Both the cardiac and skeletal muscle use a troponin-dependent mechanism for contraction, but cardiac troponin in particular has regions of specific amino-acid sequences that are different from the troponin found in striated muscles. The cardiac troponin contains three subunits: cTnC, cTnI, and cTnT, of which only cTnI and cTnT are used in clinical practice in case of cardiac muscle injury, given the higher sensitivity and specificity. Small amounts of cTnT have been found in skeletal muscle, whilst cTnI has not been identified in any other body tissue and it could be the most specific subunit of troponin for the cardiac muscle [13].

Cardiac troponin (cTn) is the gold-standard biomarker for patients with ACS and it is used to differentiate acute myocardial infarction from unstable angina [14]. Beside its role in the diagnosis of ACS, cTn also has a prognostic value for a multitude of acute and chronic diseases: chronic heart failure, acute hypotension, left ventricular strain, increased right heart strain by pulmonary embolism or by pulmonary hypertension, hypertrophic cardiomyopathy, catecholamine excess, myocarditis, toxic substances or trauma. Non-cardiac diseases such as sepsis, chronic kidney disease, hemodialysis, diabetes mellitus and stroke may also be involved in troponin value elevation [15].

cTnT can revert to a fetal isoform that can be hyperexpressed in certain diseases such as heart failure. The dying skeletal muscle can re-express fetal cTnT, which can be detected by immunoassays. Moreover, there is data showing that hereditary and acquired skeletal myopathies are associated with chronically elevated cTnT values when using high sensitivity immunoassays, probably through a cross-reaction mechanism with skeletal muscle troponin isoforms or because of the re-expression of the fetal cTnT in the dying myocite [16,17,18]. Also, extreme exercise can cause cTnT elevation, as seen in professional athletes after strenuous endurance exercises or after high intensity intermittent exercises [19].

Various neurologic disorders are associated with cardiac complications that have an impact on mortality, especially in the acute phase: aneurysmal subarachnoid hemorrhage (SAH), intracerebral hemorrhage (ICH), traumatic brain injury, acute ischemic stroke (AIS), seizures, acute hydrocephalus, encephalitis, spinal cord infarction, electroconvulsive therapy, acute motor axonal neuropathy or Guillain-Barre syndrome [20]. The mechanism implies myocardial injury by activation of the neuro-cardiac axis as a stress response to catastrophic neurological injury, leading to stress-related cardiomyopathies, including “neurogenic stunned myocardium (NSM)”, “neurogenic stress cardiomyopathy” (acute left ventricular failure in the critically ill)”, “Takotsubo cardiomyopathy” (emotional cardiomyopathy), and cardiomyopathy associated with pheochromocytoma or exogenous catecholamine administration [20]. A mildly elevated cardiac troponin value following acute brain injuries has been observed in up to 40% of SAH patients. The enzyme may accurately indicate myocardial dysfunction and other cardiovascular abnormalities. It can also be a severity indicator for short term mortality. Although CK-MB rises as well in many cases of NSM after acute brain disorders, troponin has a greater sensitivity and specificity (100% and 91% respectively) [21,22].

Regarding stroke, the American Heart Association/American Stroke Association advise cTnC assessment in every patient diagnosed with AIS, given its clinical implications and prognostic significance [23,24,25,26]. There are still uncertainties about the mechanisms by which troponin could increase after stroke. Often, patients with elevated hs-cTnT have ST segment depression and/or T-wave inversion, so the occurrence of an acute silent myocardial infarction prior to stroke has been proposed as a causal theory. The association of heart failure, hypercholesterolemia, and renal failure has also been proposed [26]. However, there are many patients with enzymatic increase but with normal electrocardiographic morphology, and with normal renal function and with no history of cardiac symptoms. Nevertheless, higher stroke severity and insular cortex involvement were significantly associated with higher cTnT levels, suggesting neurologically induced myocardial injury [27]. Insular cortical lesions can lead to loss of the central inhibitory control and an autonomic derangement with an increased sympathetic tone. Though in acute stroke the magnitude of catecholamine release is not as important as that seen in SAH, a correlation has been shown between troponin level elevation and epinephrine/cortisol levels, indicating an imbalance between parasympathetic and sympathetic nervous systems’ activity, with a high potential to induce myocardial stress [28]. Moreover, the troponin elevation is amplified if the patients with AIS have pre-existing comorbidities such as systemic atherosclerosis, hypertension, diabetes mellitus, atrial fibrillation or heart failure with a reduced cardiac output. These comorbidities are often associated with AIS, as common risk factors for both cerebrovascular and coronary arteries diseases. Also, a preexisting non-critical coronary stenosis may favor a myocardial injury when a stress condition occurs, due to the mismatch in oxygen supply and demand (Type II MI) [29].

Regarding dementia, some studies show that cognitive impairment is associated with higher levels of hs-cTnT, with a possible connection between subclinical cardiac dysfunction and subclinical brain injury. When evaluating the degree of subclinical brain injury by brain imaging, white matter lesions may indicate cerebral small vessel disease and are associated with cognitive decline and cortical atrophy [30,31]. Moreover, elevated troponin T levels (over 13.9 ng/L) are associated with periventricular and white matter hyperintensities, lacunes and cortical cerebral microinfarctions in patients with cardioembolic stroke and vascular dementia due to traditional cardiovascular risk factors (atrial fibrillation, cerebral atherosclerosis, advanced age, hypertension or low cardiac output) [32].

Although biomarkers for atrial fibrillation are not a part of current guidelines and clinical practice, several studies indicated that elevated heart rate is one of the determining factors for high cTnI levels. An increased heart rate may be associated with ischemic stress due to high oxygen demand, without the presence of coronary artery disease. Besides the fast ventricular response in AF, pre-existing heart failure with or without left atrial enlargement may lead to troponin elevation in AF. In AF patients a significant correlation has been observed between troponin values and an increased stroke risk, so elevated hsTnT may be an independent predictor of stroke [33,34].

It is unclear why patients with embolic stroke, with an undetermined source are more likely to show increases in cTnC values than patients with non-cardioembolic stroke. Although patients with an undetermined embolic stroke source do not have clear cardioembolic risk factors (such as congestive heart failure, AF or recent myocardial infarction), it is assumed that most emboli may also be of cardiac origin. Regardless of the type of impaired myocardial function, increased cTnC represents a prognostic factor in patients with acute ischemic stroke [35].

Multiple studies have shown that patients with acute ischemic stroke and elevated cardiac biomarkers have an increased risk of mortality or complications upon discharge. CTn elevations should be seen as a negative prognostic factor in the acute neurologic disorders, as cardiovascular diseases are associated with a higher long-term mortality in stroke survivors. Moreover, higher levels of hs-cTnT in AIS are associated with elevated levels of thrombo-inflammatory molecules and can predict NIHSS (National Institute of Health Stroke Scale) worsening with a progressive neurologic deficit. Furthermore, a correlation has been reported between elevated troponin levels and a poor neurological outcome after AIS on hospital discharge [36,37].

### 2.3. Creatine Kinase (CK) and the CK-MB Isoform

Creatine phosphokinase (CPK or CK) is the enzyme that catalyzes the conversion of creatine into phosphocreatine, converting ATP to ADP, thus representing an energy reservoir for ATP regeneration. The enzyme is abundant in the cytosol and in the mitochondria of cells that require substantial amounts of ATP, like those of the brain, skeletal muscles, and the heart [38]. The muscle type (M chain) and the brain type (B chain) of CK present in the cytosol may produce variants like heterodimers (CK-MB) or homodimers (CK-MM) and (CK-BB) that can be released in the bloodstream secondary to cell destruction. CK-MB is mostly present in the cardiac muscle, as the myocardium has about 17% to 59% CK-MB isoenzyme. Thus, CK-MB is a more specific indicator for myocardial muscle damage. CK-MM is a principal component in skeletal muscles, while CK-BB is present in smooth muscle and most non-muscle tissues such as the brain [39].

Skeletal muscles contain on average 5–6% of CK-MB, although there are some muscles with up to 20% CK-MB (type 1 muscle fibers), or up to 40–50% CK-MB (regenerating muscles fibers that revert to an embryonic enzyme pattern). Muscular damage will increase total CK activity in the bloodstream, with an increase of CK-MB isoenzyme as well, but usually less than 6% of total CK. Higher percentages of CK-MB may appear after acute muscle injury, surgical interventions, in marathon runners and chronic neuromuscular diseases due to the regenerating fibers. The normal CPK level is considered to be 20 to 200 IU/L. Still, there are variations of normal CK levels and CK-MB percentages that differ significantly by sex, race or age, possibly due to differences in muscle mass and inherited differences in the sarcolemma permeability. Children have higher percentages of CK-MB (up to 26% CK-MB of total CK), perhaps due to the growing process. Low levels of CPK may be present in patients with connective tissue diseases such as rheumatoid arthritis or systemic lupus erythematosus. Reduced physical activity as in elderly bedridden patients will also result in low levels of CPK. Also, there is a small reduction in CK levels as people age [38,39,40,41,42].

Many situations are associated with an increase of CK levels. Intense physical activity may increase CK levels up to 30 times the normal value within the first 24 h, as do intramuscular injections or electromyography. Moreover, increased values are also found in hypothyroidism, metabolic or oncological disorders, following surgery, during pregnancy, in acute renal failure, mediastinal radiotherapy, pneumonia, lung infarction, colonic infarction, metastatic carcinoma, sepsis, shock, viral diseases, or due to treatment (daptomycin, statins, antiretrovirals) [43,44].

CK and CK-MB have been used for a long time as the most common biomarkers for investigating patients with acute coronary syndromes (ACS), as they rapidly increase after the onset of symptomatology. In these patients, total CK enzyme values reach a peak after 12–24 h, while CK-MB has maximum values at a 10–18 h interval. It is important to measure their levels repeatedly, every 6–8 h in the first 24 h, in order to capture the enzymatic dynamics. Patients with rapidly rising and falling levels of CK-MB, exceeding the normal limits, must be considered as having ACS until proven otherwise. The high level of CK-MB released as a result of skeletal muscle damage persists for a longer period of time than after a myocardial injury. Compared to other cardiac enzymes, CK-MB is usually normal in sepsis, malignancy and renal failure. A CK-MB relative index < 5% (ratio of CK-MB to total CK) is usually correlated with a skeletal muscle source, while an index of >5% is consistent with a cardiac one. However, in cases of chronic skeletal myopathies, the percentage value may increase [45,46].

Among cardiovascular diseases, CK-MB does not only increase in acute coronary syndromes, but also in other types of myocardial injuries such as trauma, arrhythmias, congestive heart failure, myocarditis, pulmonary thromboembolism, shock or invasive maneuvers such as cardiac catheterization or heart surgery [45]. 

False elevations in CK-MB may occur in the presence of atypical CK isoforms, macrokinases or adenylate kinase plasma activity, due to certain abnormalities that occur in the CK isoenzymes. The most common cause is the formation of the so-called macro-creatine kinase complexes (macro-CK) especially if the immune-inhibition assay method is used for measurement. A ratio of total CK/CK-MB > 50% is suggestive for the presence of macro CK complexes, while an inversed ratio is highly specific for macro-CK. There are two types of macro CK: macro CK type 1 is mostly found in patients with hypothyroidism, autoimmune diseases, myositis, cardiovascular disease and, apparently, even in healthy individuals and occurs via an antigen-antibody type reaction between CK-BB and IgG or between CK-MM and IgA; macro CK type 2 is found in severely ill patients, particularly in those with malignancies (most commonly colon or prostate cancer) and severe liver disease [47,48,49].

Statin therapy is widely recommended as a lipid-lowering treatment or for their pleiotropic effects, in hypercholesterolemic patients, after myocardial infarction or after stroke. Some of the known adverse effects of statins are muscle-related disorders, like myalgia, muscle weakness, rhabdomyolysis or, rarely, an immune-mediated necrotizing myopathy. Up to 5% of the patients develop CK elevation of up to 10 times the normal upper limit, especially when initiated or after a dosage increase. The risk for adverse effects increases if the patient has liver or kidney disease, untreated hypothyroidism or receives a concurrent drug that inhibits cytochrome P450–3A4 (CYP3A4) such as cyclosporin, fibrates, calcium channel blockers, protease inhibitors, or warfarin; grapefruit juice has a similar effect [50,51]. 

A particular interest is the increase of the CK-MB isoform in non-cardiac pathology, especially in acute neurological disorders such as stroke. 54% of these patients have increased CK-MB on the first day after stroke, while 72% of them may have increased CK values [52]. Increased intracranial pressure or insular disinhibition leads to the release of catecholamines by activating the vegetative nervous system after an ischemic stroke, which can lead to tachycardia, coronary vasospasm and also direct myocardial toxicity through increased intracellular calcium levels. There are also ECG changes in about 32% of patients, possibly due to autonomic neural stimulation from the hypothalamus or elevated circulating catecholamines that can lead to the misinterpretation of the increased enzymes’values, without necessarily suggesting an acute coronary injury. Insult of the insular cortex is particularly associated with cardiac complications after ischemic stroke, by involving the autonomic centers and the sympathetic activation. Another mechanism of increased CK and CK-MB values in stroke concerns the metabolic origin in patients with inadequate post-stroke nutrition, where negative caloric balance leads to the release of caloric energy from the skeletal muscle [44,53].

In the differential diagnosis of increased CK levels, neuromuscular pathologies should also be taken into account, as CK plasma level is increased by muscular damage, necrosis or regeneration of myocytes. Myotonic dystrophies type 1 (Steinert) or type 2 may be accompanied by increased values of this enzyme. In patients with type 2 myotonic dystrophy, the increased CK values were observed in 83% of cases and the levels were higher than in type 1 myotonic dystrophy. Asymptomatic women, carriers of the defect for Duchenne or Becker muscular dystrophy types, may sometimes be diagnosed by elevated serum CK levels of 25 to 200 times higher than normal. However, in the slowly progressive forms of dystrophy the CK levels may be normal. A persistent increase in CK values is seen in many types of genetically determined muscular dystrophies, with different variants of abnormal dystrophin or membrane proteins. Very high values are found in Duchenne muscular dystrophy and in conditions where severe muscle necrosis occurs, such as acute polymyositis, rhabdomyolysis associated with malignant hyperpyrexia or in the metabolic myopathies. Although both the Duchenne and Becker forms of muscular dystrophy are associated with a form of cardiomyopathy similar to myocarditis that lead to cell death, fibrosis and heart chamber dilatation, the proper cardiac injury biomarker for diagnosis of cardiomyopathy should not be CK-MB, but rather troponin, since in most muscular dystrophies, given the chronically high myocyte turnover, the gene for the B subcomponent of creatine-kinase can be de-repressed, thus resulting in higher levels of CPK-MB isoenzymes with skeletal muscle origin that can lead to misinterpretation [54,55,56,57].

CK values may increase in patients with rapid muscle denervation such as amyotrophic lateral sclerosis (ALS), but also in progressive spinal muscular atrophy when the disease progression is sufficiently rapid. CK expression seems to be higher in astrocytes and oligodendrocytes compared to neurons. So, peripheral neuropathies or radiculopathies of different etiologies can lead to increasing levels of CK. Muscle weakness is a feature of most neuropathies and the degree of motor deficit is proportional to the number of axons of motor neurons affected. 27% of patients with chronic inflammatory demyelinating polyneuropathy (CIDP) had elevated CK levels through axonal degeneration. In Guillain-Barre syndrome there is an acute motor paralysis that may cause changes in CK values. A possible explanation would be that axonal loss, especially in the proximal musculature, may alter the integrity of the muscle membrane. Charcot-Marie-Tooth disease, a hereditary sensory-motor polyneuropathy, may also be accompanied by increased CK values [58,59].

Central neurological pathology may also be involved in altering the values of this biomarker. CK could be a useful biomarker with high specificity in the diagnosis of generalized tonic-clonic epileptic seizures in emergency departments. Increased CK values have been observed in these situations as compared to the psychogenic nonepileptic seizures or the vasovagal syncopes [60]. As well, meningitis frequently increases CK or the CK-BB isoenzyme [61].

In several studies, creatine proved to have neuroprotective properties by defending neurons against toxic metabolites and counteracting glucose and serum deprivation [62,63]. CK is essential for maintaining the cellular homeostasis of creatine and it is an important regulator of cellular energy regeneration in the brain [64,65]. In Alzheimer’s disease a substantial decrease in total CK activity has been observed by an inactivation of CK due to reactive oxygen and nitrogen species (BB-CK activity decreased by 86%, and the expression level of CK was reduced by less than 14%) [66]. Also, decreased activity of CK was observed in epilepsy, schizophrenia, and maniac-depressive psychosis, but to a lesser degree [66,67,68]. Patients with Huntington’s disease and Pick disease may also have an impaired energy metabolism due to decreased CPK activity [69,70].

Some studies suggest that the down-regulation of CK-MB by a mutant gene is a key element in the pathogenesis of Huntington’s disease, leading to neuronal dysfunction. Therefore, increasing CK-MB expression might be a promising treatment option, apart from the classic dietary supplementation with creatine [71].

An increase in CK-MB levels was also observed in some studies in childhood benign paroxysmal vertigo attacks, suggesting a possible pathophysiological association [72,73].

### 2.4. Galectin-3 

Galectin-3 (Gal-3) is a β-galactoside-binding lectin with important roles in intercellular and cell-matrix interactions, cell growth and differentiation, immunity, inflammation, angiogenesis, fibrosis and apoptosis. Due to its broad involvement in multiple pathological pathways, the research of recent years included this biomarker in the study of various pathologies: cardiac, nephrological, autoimmune, neurological and glioma tumorigenesis [74].

In HF, given its role in inflammation, fibrosis and cardiac remodeling, it was proven as a useful biomarker for prognosis and risk stratification. A persistently elevated plasma value of Gal-3 in the general population was associated with an increased risk for HF [75]. Also, in patients already diagnosed with HF, Gal-3 could predict adverse cardiovascular outcomes and all-cause mortality [76,77,78]. The correlation between the Gal-3 levels and the NYHA class further supported its prognostic value in HF [79]. As natriuretic peptides reflect the cardiac volume status and Gal-3 the cardiac remodeling, they have a cumulative prognostic value when measured in a multimarker approach. Therefore, it is recommeded to use Gal-3 as an additive biomarker to natriuretic peptides for prognosis evaluation [80]. The multimarker approach in heart failure for prognosis evaluation is part of ACC/AHA guidelines, but it is not yet recommended in the ESC guidelines [81,82]. Positive results for Gal-3 as a prognostic biomarker were found in patients with a previous myocardial infarction, as it showed the capacity to improve risk stratification in those patients [83,84].

By detecting early myocardial dysfunction, plasma Gal-3 proved to be an equally useful diagnostic tool in patients with HF with preserved ejection fraction, a challenging clinical situation if NT-proBNP values are within the normal range [79,85]. As a biomarker of fibrotic degeneration and inflammatory conditions, it was studied in acute myocarditis following a viral infection. The results showed that high levels of Gal-3 were present even in the presymptomatic stages, which may be useful in establishing an early diagnosis of myocarditis [86].

Knowing the pathophysiological implication of Gal-3 in the development of HF, this biomarker may have a potential for future targeted therapies [87].

In many neuropathological diseases (traumatic brain injury, ischemic insult, encephalitis), Gal-3 was associated with microglial activation and recruitment of macrophages that were involved in phagocytosis of degenerated myelin. The removal of degenerated myelin by activated microglia and macrophages is essential for the regeneration of the myelin sheath after an axonal injury. At the same time, microglia, as the main source of pro-inflammatory molecules in the brain (cytokines, chemokines, reactive oxygen or nitrogen species), may create a neurotoxic environment if it has a sustained activity. In the first 24 h after a cranio-cerebral trauma event, an increased release of Gal-3 in the cerebrospinal fluid was noticed, which further promoted neuronal loss [88].

Through a similar mechanism, Gal-3 contributes to the progression of neurodegenerative processes like Alzheimer’s disease [89]. Elevated serum and cerebrospinal fluid levels of Gal-3 were found in both Alzheimer’s and Amyotrophic Lateral Sclerosis (ALS) patients, with a positive correlation with the MMSE (Mini-Mental State Examination) score, plasma Gal-3 being able to predict the neuropsychological decline [89,90].

Laboratory studies on animal models showed that the Gal-3 induced deficiency may reduce the severity of multiple sclerosis, suggesting that Gal-3 plays an important pathogenic role in multiple sclerosis and could be a therapeutic target for autoimmune demyelinating diseases [91]. In Huntington’s disease, before motor impairment, an amplified Gal-3 expression with constant increased plasma levels throughout the disease progression was noted [92]. Also, serum Gal-3 proved to be efficient in the diagnosis and the prognostic assessment of Parkinson’s disease [93].

Some clinical studies have indicated that serum Gal-3 levels may be useful for the prognosis evaluation in cerebrovascular diseases such as stroke, intracerebral hemorrhage or subarachnoid hemorrhage caused by an aneurism. In view of the fact that Gal-3 is involved in the inflammatory cascade within neurons, higher serum levels correlate with unfavourable outcomes in these patients [94,95,96].

It may also be helpful in assessing the clinical outcomes in newborn infants with birth asphyxia, as elevated values of Gal-3 in the cerebrospinal fluid was associated with global brain ischemia and a more severe clinical evolution [97].

### 2.5. Soluble Form of the ST2 Protein (sST2)

The soluble form of the ST2 protein (sST2) is a promising biomarker in heart failure [98]. ST2 is a member of the interleukin-1 (IL-1) receptor/Toll-like receptor superfamily. The signaling pathway involving the interaction between the transmembrane form of the ST2 protein (ST2 L) and its ligand, IL-33, plays a cardioprotective role by inhibiting the inflammatory response and fibrosis [99]. On the other hand, soluble ST2 competes with ST2-L for IL-33 binding, consequently diminishing the cardioprotective effects of the signaling pathway, promoting myocardial fibrosis and cardiac remodeling [99]. Thus, patients with high serum concentrations of sST2 associate ventricular remodeling and may develop heart failure [100,101]. In patients with heart failure, sST2 values were higher in the acute setting and in patients complaining of dyspnea [102]. Also, it was identified as a useful predictor for one-year mortality in heart failure patients [103]. Taking into consideration its rapid level fluctuations in the blood, sST2 was also studied, with good results, as a biomarker for monitoring and guiding therapy in heart failure patients [104,105]. As it seems to be less influenced by other patient characteristics such as age, body weight, anemia, and renal impairment, sST2 may provide additional information in combination with natriuretic peptides in HF patients, and the ACC/AHA guidelines included sST2 in a multimarker approach for prognosis evaluation [81,101,102].

In neurological pathologies, sST2 proved to be efficient, especially in cerebrovascular diseases. Plasma levels of sST2 measured early after the rupture of a cerebral aneurysm had a predictive role for delayed cerebral ischemia, functional neurologic outcome and mortality. Increased levels of sST2 in patients with subarachnoid hemorrhage were associated with a shift towards a more pro-inflammatory immune cell population, which had a negative impact on the outcome [106]. As a prognostic marker, it was noticed that sST2 levels were associated with neurophysiologic changes on continuous electroencephalography, such as new or worsening epileptiform abnormalities after an episode of subarachnoid hemorrhage [107].

In patients with ischemic stroke, soluble ST2 can independently predict the 90-day outcome, hemorrhagic transformation and mortality, as a result of a possible link between neuroinflammation and secondary injury after stroke [108]. This risk is further increased by the alteration of the blood-brain barrier with the crossing of pro-inflammatory molecules, which lead to post-ischemic neuroinflammation [105]. sST2 is also studied as a long-term prognostic marker after an episode of ischemic stroke or transient ischemic attack [109]. Also, it can predict moderate to severe cerebral-cardiac syndrome after an ischemic stroke (coexisting NIHSS > 8 and left ventricular ejection fraction < 60%), likely due to its role in inflammatory cardiovascular diseases and myocardial fibrosis [110]. Furthermore, sST2 proved its efficiency in detecting subclinical brain injury after an incident stroke [111].

There is data indicating that serum sST2 has significantly higher levels in the early phase of Alzheimer disease patients (with mild cognitive impairment), with a reduced IL-33 expression. The pathogenesis of Alzheimer disease may involve an impaired IL-33/ST2 signaling, with the inhibition of β amyloid phagocytosis and clearance by microglia, which promotes chronic neuroinflammation and amyloid plaque deposition [112]. Amyotrophic lateral sclerosis and myasthenia gravis were, as well, associated with high levels of sST2 [113,114].

### 2.6. Lysophosphatidylcholine

Lysophosphatidylcholine (LPC) may be associated with the pathogenesis and correlated with the prognosis of cardiovascular diseases but also with several neurological disorders. LPC represents a lipid biomolecule derived from the turnover of phosphatidylcholine via phospholipase A2 or produced on the surface of high-density lipoprotein (HDL) and low-density lipoprotein (LDL) via lecithin-cholesterol acyl transferase (through the addition of fatty acids to free cholesterol). Overproduction or impaired degradation of LPC increases the LPC levels in the circulation and the concentration of LPC inside the LDL and the oxidized LDL molecules, so it is significantly involved in the pathogenesis of atherosclerosis [115,116].

Lysophosphatidylcholine is known to be a bioactive proinflammatory lipid that affects arterial distension, induces endothelial dysfunction, increases oxidative stress and promotes fatty acid-induced insulin resistance [117]. Consequenly, LPC is associated with the development of many cardiovascular diseases, such as myocardial infarction, angina and heart failure [118,119,120,121]. Also, increased plasma levels of LPC and LDL were found in patients with familial hypercholesterolemia and familial combined hyperlipidemia [122].

Many brain diseases such as multiple sclerosis, stroke, schizophrenia, depression, Down syndrome and autism are considered demyelinating disorders. Oligodendrocytes are myelin-producing cells that also support axon survival and function, preventing neurodegeneration [123]. Besides having pro-inflammatory properties and damaging effects on the vascular endothelium, LPC also alters the physiology of oligodendrocytes, induces demyelination, mediates pericyte loss and alters the neurovascular barrier, leading to neurodegenerative diseases [124]. The process of axon demyelination is caused by the accumulation of excess LPC into the central nervous system, mainly into the oligodendrocyte cell membrane, which will increase permeability and therefore induce necrotic death [124]. Increased circulating levels of LPC are caused by a variety of physiological and pathological conditions: increased degradation of phosphatidylcholine by the lipoprotein-associated phospholipase A2 (Lp-PLA2) or by the lecithin-cholesterol acyltransferase, LPC overproduction induced by hypoxia, decreased albumin-LPC binding caused by hypoalbuminemia, decreased LPC degradation by lysophospholipases [121,122,123,124,125,126,127].

Lp-PLA2 is the main enzyme involved in the LPC metabolism, and its overactivity may lead to increased levels of LPC, which may trigger neurological and cardiovascular disorders. There is increasing clinical evidence suggesting that Lp-PLA2 activity is correlated with plaque instability and acute coronary syndromes [128]. Also, Lp-PLA2 may be a prognostic factor for ischemic stroke severity, as it was observed that patients with high Lp-PLA2 levels had more severe symptoms after stroke and earlier neurological deterioration [129]. Lp-PLA2 in the early stages of aneurysmal subarachnoid hemorrhage might be a new predictive biomarker for the occurrence of delayed cerebral ischemia [130].

The complex mechanism by which the LPC molecule manifests its effects it is not yet fully understood, and more studies are necessary for its implementation in clinical practice.

### 2.7. Copeptin

Copeptin (also known as CT-proAVP) is an aminoacid derived from the C-terminal portion of the arginine vasopressin precursor. Copeptin is released into the circulation mainly from the pituitary gland in cases of hypotension or hemodynamic stress [131].

Copeptin was proven to be a valuable sensitive biomarker in the diagnosis of acute coronary syndromes, as its levels were elevated in the early stages after the onset of an acute myocardial infarction, with a negative predictive value between 97% and 100% [132]. However, copeptine is a non-specific biomarker compared to troponine or CK-MB (with high cardio-specificity), which supports the recommendation that it be used as an additional biomarker in a multimarker approach [133]. The full mechanism by which such rapid elevations of copeptin levels occur is still unknown. However, it is believed that both myocardial injury and hemodynamic stress may lead to a rise in copeptin levels. In addition to its diagnostic value in ACS, copeptin proved to be efficient as a prognostic biomarker, being correlated with the 1-year mortality and major complications after an acute coronary event [134]. Studies showed that copeptin levels were correlated with the size of the myocardial infarction and the degree of left ventricular dysfunction and ventricular remodeling [130,135]. Also, in patients with heart failure, copeptin was identified as a valuable prognosis biomarker, being able to predict adverse outcomes and mortality [128,129].

Part of the endocrine systems response to stress, copeptin levels are also influenced by cerebrovascular ischemic events. The main cause for the early release of copeptin after stroke is post-stroke cerebral edema [136]. Similar to myocardial infarction, copeptin levels correlated well with stroke size [132]. Copeptin had different values in patients with ischemic stroke and transient ischemic attack, which support its ability to distinguish between these two entities [137]. Furthermore, copeptin levels predicted unfavorable functional prognosis and mortality in stroke patients. An improved prognosis accuracy was observed after adding copeptin measurements to the NIHSS or ABCD2 score, compared to using these scores alone [138,139].

Generalized convulsive seizures (GCS) generate a hyperadrenergic state that may trigger cardiac complications, such as ST segment or T-wave abnormalities, life-threatening arrhythmias, QT interval changes or stunned myocardium. According to recent studies, copeptin increases considerably after most GCS (94% of tested seizures), even in the absence of cardiac complications [140].

Copeptin has also been studied for its efficacy in the differential diagnosis of headache in the Emergency Department. The results showed that copeptin can differentiate between serious secondary headaches (defined by a neurologic disease requiring immediate treatment) and benign forms of headaches [141].

In newly diagnosed untreated multiple sclerosis patients, plasma levels of copeptin and cortisol are correlated with the patients’ clinical condition, with higher values in overweight/obese patients [142].

### 2.8. S100B

S100B is a Ca2+ -binding protein, recognized as a reliable biomarker for active neural damage and used as a parameter of glial activation/glial death in many disorders of the central nervous system (CNS) that lead to brain damage. It is abundantly found in glial and Schwann cells in the central and peripheral nervous system, being also present in other neural populations. It plays important roles in the development and protection of CNS though its many intra- and extracellular functions (mediating neurotrophic activity, neuronal electrical activity or regeneration of neural cells in the peripheral nervous system). Also, S100B has functions attributable to neuroinflammation, as over-expression/administration induces a worsening of the disease and the deletion of the gene or its inactivation produces disease amelioration [143,144,145].

The S100B levels/distribution in the nervous tissues seems to be directly related to the progress of chronical neurodegenerative diseases (Parkinson’s disease, Alzheimer’s disease, amyotrophic lateral sclerosis, multiple sclerosis), perinatal/congenital disorders (spinocerebellar ataxia-1, Down syndrome) or psychiatric disorders (schizophrenia) [146].

Given its immediate release after an acute cerebral event, such as traumatic brain injury or stroke, it is considered to be a valuable neurobiomarker (a “troponin” of the brain) for early diagnosis [147]. Moreover, it may have an important prognosis value, as S100B proved to have the ability to show the extent of hypoxic ischemic brain injury after cardiac arrest [148]. In patients with sleep obstructive apnea syndrome, S100B was studied as a biomarker of structural brain damage [149].

Although it is considered one of the most specific and sensitive neurobiochemical markers, recent studies show that S100B may have other extracerebral sources, besides the astroglia and Schwann cells. Several studies mentioned the increase of serum S100B levels after cardiac interventions such as catheter ablation for atrial fibrillation, cardiopulmonary bypass or other cardiac surgical interventions such as vascular by-pass [150,151,152,153]. The main hypothesis was that the protein elevation was secondary to asymptomatic brain injury, as S100B is considered to be specific for the brain. However, there were no statistically significant correlations with the imaging findings on the DW-MRIs (Diffusion-weighted magnetic resonance imaging) conducted after the cardiac interventions. Subsequent studies have shown that S100B may also have a cardiac origin, as the myocardium is known to possess a network of high-density nerve fibers responsible for its autonomic control (several types of neurons, glial cells, and interconnecting fibers that control the physiological cardiac cycle) and specialized cells with similar characteristics to neurons through the excitoconduction system (automatism, conductivity). Collateral damage of this delicate intracardiac neural network may be the source of changes in S100B plasma concentrations after catheter ablation for atrial fibrillation, as it is suggested by a study that showed S100B expression in cardiac glial cells throughout the intracardiac microsystem using immunohistolochemical staining [154]. Furthermore, patients with higher S100B release had lower rates of atrial fibrillation relapse during follow-up, as S100B may contribute to the sprouting of local neurons, diminishing action potential firing by reducing neuronal electrical activity, and increasing neurite growth. Meanwhile, as some studies detected the increase of another neurobiomaker after such interventions (neuron specific enolase), minimal neuronal injury should not be fully excluded in these cases [155].

There is increasing evidence with experimental rodent models that S100B may play a role in the adverse ventricular remodeling process after myocardial infarction, its production being induced as a response to α1-adrenergic stimulation [156]. S100B contributes to the remodeling process by enhancing the release of the potent endothelial-cell specific mitogen vascular endothelial growth factor (VEGF) via RAGE ligation, which induces myofibroblast proliferation [157]. 

Also, by activating a transcriptionally inducible form of nitric oxide synthase (iNOS), S100B plays a role in modulating the apoptosis process of cardiomyocytes during myocardial infarction [158]. These data suggest that S100B could be a potential novel therapeutic target for patients with myocardial infarction.

As the expression of S100B in the heart tissue is induced by the remodeling processes, S100B may be a promising biomarker for establishing the diagnosis and prognosis in heart failure patients. A study conducted on patients with dilated cardiomyopathy found a positive correlation between S100B and NT-proBNP serum levels [159]. The study excluded other conditions that could influence S100B serum levels. 

Another recent study shows that S100B protein accumulates in injured cardiomyocytes in drug-overdose sudden death cases, with protein immunoreactivity observed in the cytoplasm of cardiomyocytes. Therefore, S100B immunopositivity may be used as a new screening tool for the postmortem diagnosis of overdose-related cardiac death [160].

### 2.9. Myeloperoxidase (MPO)

Myeloperoxidase (MPO) is an enzyme stored in the azurophilic granules of neutrophilic leukocytes, also present in monocytes and tissue macrophages, being released in the extracellular space during an inflammatory process. This enzyme has major pro-oxidative and pro-inflammatory properties, contributing to the process of atherogenesis through its involvement in the oxidation of low-density lipoproteins (LDL) and the increased predisposition to rupture of atheroma plaques. MPO leads to the formation of a highly reactive oxygen species, hypochlorous acid (HOCl), which interacts with LDL and contributes to atherosclerosis by multiple mechanisms: the stimulation of the release of chemokines from monocytes and the chemotactic migration of neutrophils, the inactivation of the lysosomal proteases of macrophages leading to intracellular lipid accumulation and endothelial dysfunction, the stimulation of leukocyte adhesion and migration in the subendothelial space and the increase of platelet reactivity. MPO also induces oxidative modifications of HDL, attenuating its anti-atherogenic properties. There was a positive correlation between serum MPO concentration and the presence of coronary heart disease, and also with its severity. Thus, MPO has higher values in patients with myocardial infarction as compared to those with stable angina, and the concentration depends on the degree of coronary artery stenosis on the angiography. It seems that MPO may also have a predictive role for major acute cardiac events, increased values being related to the neutrophils’ and macrophages’ activation before the atheroma plaque rupture. MPO values may be increased up to two hours after the onset of symptoms, possibly even before the myocardial injury occurs. MPO could also predict long-term mortality in patients with coronary disease [161,162,163,164].

Neuroinflammation is an important component in neurological pathology and MPO could be an essential biomarker in investigating certain dysfunctions in this field. MPO can compromise the functionality of the blood-brain barrier by altering the expression of cytokines and chemokines. Also, it has been observed that polymorphisms of the MPO promoter region, which lead to an increased expression of the biomarker, are associated with a higher incidence of Alzheimer’s disease, a pathology caused by beta-amyloid deposits in the brain. MPO can be identified mainly at the level of amyloid plaques in the neurons of the neocortex and hippocampal region [165].

MPO may also be involved in the progression of Parkinson’s disease, characterized by the presence of Lewy bodies in the pigment cells of the black substance and other pigmented nuclei. This contributes to the activation of microglia and the release of MPO, which leads to oxidative stress and neuronal degeneration [165].

Increased serum MPO values were also observed in patients with stroke. On the murine models, increased MPO values persisted for 21 days after stroke. When MPO is inhibited, neurogenesis can be stimulated by differentiation, proliferation, migration, and survival of new cells. Thus, MPO inhibition may represent a therapeutic perspective in post-stroke patients, improving neuronal functionality [165].

MPO is considered an important biomarker in major depressive disorder. The symptoms of depression do not appear to be correlated with MPO, but both inflammation and depression have common pathophysiological pathways. Other inflammatory markers were found in depressive disorder, but their values were not as high as MPO [165].

MPO is activated by macrophages and microglia present at the level of demyelination plaques in multiple sclerosis. Thus, it contributes to the release of the cytotoxic compound HOC1 which further damages the myelin sheath [165].

Epileptic seizures are caused by neuronal hyperexcitability and contribute to the release of free radicals. The activation of macrophages and microglia results in the release of MPO and subsequently of HOC1, which, by cytotoxicity, leads to epileptogenesis. The brain is more vulnerable to inflammation and oxidative stress during epileptic seizures [165]. MPO contributes to the spread of inflammation in certain neurological pathologies, resulting in tissue damage. Thus, MPO inhibition may represent a therapeutic perspective in epilepsy and neurodegenerative diseases [165].

### 2.10. Growth Differentiation Factor-15 (GDF-15)

Growth differentiation factor-15 is a cytokine produced mainly in the cardiomyocytes, adipocytes, macrophages, endothelial cells and smooth vascular musculature, being expressed especially in conditions of tissue injury, inflammation and mechanical or oxidative stress [166,167]. Its effect is cardioprotective, increased levels reflecting the process of healing the lesions [168]. GDF-15 levels were associated with cardiovascular pathologies such as heart failure, hypertrophy, atherosclerosis, endothelial dysfunction and recurrent myocardial infarction [167,168]. GDF-15 levels increase in just a few hours after a myocardial infarction and remain elevated for a few days. The values are significantly higher in patients with acute myocardial infarction compared to those who have unstable angina or non-cardiac chest pain. Also, GDF-15 increases the predictive value of the GRACE (Global Registry of Acute Coronary Events) score in patients with NSTEMI myocardial infarction, even more than NT-proBNP and may thus be useful in the risk stratification for choosing an invasive treatment [166]. Also, GDF-15 seems to improve the HAS-BLED risk score, with a strong association between the values of this parameter and the bleeding risk [169]. Increased levels of GDF-15 are also predictive for all-cause mortality in patients with a non-ischemic etiology. The values of this biomarker increase as heart failure advances and could be useful in asymptomatic patients with a progressive disease [168].

GDF-15 is also secreted by erythroblasts as they mature and is one of the proteins involved in hepcidin regulation and iron homeostasis. Situations associated with ineffective erythropoiesis (thalassemia syndromes or sickle cell syndromes), as well as other conditions, such as inflammation, acute injury, cancer, and chronic kidney disease, were identified as modifiers for serum GDF-15 concentrations [170,171,172].

In the neurological pathology, this biomarker is not well researched as of now. However, it has been observed that it may have a role in stroke patients. GDF-15 promotes angiogenesis under ischemic conditions, which may explain the increased levels in patients with ischemic stroke [173]. In those who had thrombolysis or thrombectomy, increased levels of GDF-15 on admission were associated with an increased risk of death at 3 months. Values decrease within 24 h after stroke, but sometimes they can persist for up to 7 days. Also, it was observed that GDF-14 values are in accordance with the NIHSS score, an increased level being associated with a higher severity of symptomatology and therefore with a greater ischemic injury [174]. In hypertensive patients, GDF-15 is an independent predictor for the occurrence of a first stroke, especially an ischemic one [173].

## 3. Conclusions and Future Perspectives

Most of the biomarkers used and studied in cardiovascular diseases proved their usefulness in many neurological conditions. This narrative literature review highlighted the various situations that require a differential diagnosis between the two types of pathologies (cardiac and neurologic). As it is difficult to identify a biomarker with perfect specificity and sensitivity for a particular pathology, the clinical context and other types of investigations are always useful for a correct diagnosis. However, biomarkers can be extremely important in assessing the prognosis, especially in cardiac and neurological pathologies, as they frequently interfere and influence each other. Thus, further research is needed for discovering better and more specific biomarkers.

## Figures and Tables

**Table 1:** Potential role of biomarkers in cardiovascular and neurological pathologies.

	Biomarker	Cardiovascular Diseases Roles		Neurology Pathologies Roles	
		Diagnostic	Prognostic	Diagnostic	Prognostic
*Used in Clinical Practice*	NT-proBNP	+	+	−	+
	Troponins	+	+	−	+
	CK, CK-MB	+	+	+	+
	Galectin-3	+	+	−	+
*Future Potential Use in Clinical Practice*	sST2	+	+	−	+
	LPC	+	+	−	+
	Copeptin	+	+	+	+
	S100B	−	+	+	+
	MPO	+	+	+	+
	GDF-15	+	+	+	+

NT-proBNP—N-terminal pro-natriuretic peptide type B; CK—creatine kinase; CK-MB—creatine kinase isoform MB; sST2—soluble form of the ST2 protein; LPC—Lysophosphatidylcholine; MPO—Myeloperoxidase; GDF-15—Growth differentiation factor-15.
